# Disseminated tuberculosis in a cow and a dromedary bull‐camel in Zamfara State in Nigeria

**DOI:** 10.1002/vms3.132

**Published:** 2018-10-25

**Authors:** Ibrahim Ahmad, Caleb A. Kudi, Abdullahi A. Magaji, Yusuf Yakubu, Mansur D. Salisu, Samuel Shuaibu, Zaharadden M. Daninna

**Affiliations:** ^1^ Directorate of Animal Health and Livestock Development Gusau Zamfara Nigeria; ^2^ Department of Veterinary Medicine Faculty of Veterinary Medicine Ahmadu Bello University Zaria Zaria Kaduna Nigeria; ^3^ Department of Veterinary Public Health and Preventive Medicine Faculty of Veterinary Medicine Usmanu Danfodiyo University Sokoto Nigeria; ^4^ National Animal Production Research Institute Ahmadu Bello University Zaria Zaria Kaduna Nigeria; ^5^ Federal Medical Center Gusau Gusau Zamfara Nigeria

**Keywords:** disseminated tuberculosis, cow, dromedary camel, Nigeria

## Abstract

In 2017, cases of generalized tuberculosis (TB) were identified in a cow and a bull‐camel, slaughtered at the Gusau abattoir in Zamfara State Nigeria. The objective of this study was to identify the cause of the gross pathology on the account of disseminated lesions widely distributed in different body parts of the animals. Tissue samples were analysed using Ziehl–Neelsen (ZN) stain and region of difference (RD) deletion typing. Results obtained by laboratory investigations revealed infection with acid‐fast tubercle bacilli in affected tissues from the cow and the dromedary bull‐camel. This study presents the first molecular evidence of *Mycobacterium bovis* infection in a Nigerian dromedary camel, demonstrated the ease of identification of the acid‐fast bacilli using molecular method which improves diagnosis and can aid epidemiological studies.

## Introduction

Bovine tuberculosis (bTB) is an important livestock infectious disease raising public health and economic concerns. It is caused by bacteria belonging to the *Mycobacterium tuberculosis* complex (MTBC), which consists of closely related species, namely; *Mycobacterium (M.) bovis*,* M. caprae*,* M. tuberculosis*,* M. africanum*,* M. canettii*,* M. microti* and *M. pinnipedii* (Forrellad *et al*. 2013). BTB is a chronic disease characterized by a gross pathology named tubercles mostly found in the lungs and lymph nodes. However, it occasionally affects other tissues such as the pleura, lactating glands, gastrointestinal and urogenital tracts. Infection is transmitted between animals through infective aerosols, direct contact, suckling and sharing of water and feeds (El‐Sayed *et al*. 2016).

In most regions of Africa, bTB remains prevalent and preventive measures such as surveillance activities and milk pasteurization are not extensively practised (Franco *et al*. 2017). The disease can be transmitted to humans through consumption of contaminated raw milk, untreated dairy products, meat with tuberculous lesions containing *M. bovis* and inhalation of contaminated aerosols from infected animals. Few laboratories in developing countries have systems in place to identify *M. bovis*, and thus most mycobacterial species that cause infection are not properly differentiated (Franco *et al*. 2017). As the only control programme to prevent exposure of high‐risk individuals such as abattoir workers and consumers of animal products to zoonotic TB, food animals slaughtered in Nigerian abattoirs undergo post‐mortem examination. In carcasses found with bTB‐like lesions, the affected tissues are trimmed off partially or wholly condemned, but rarely samples from diseased animals are investigated further unless for research purposes. In the event of suspected cases of bTB, it would be more valuable to be able to prove infection by identifying the species of acid‐fast bacilli (AFB) involved, so as to lessen the risk of the disease transmission. This study was formulated to describe cases of disseminated tuberculosis in a slaughtered cow and a dromedary bull‐camel, identified during meat inspection.

## Case presentation and pathological examination

In January 2017 during meat inspection activities, generalized bTB lesions were detected in a slaughtered cow aged 2–3 years at Gusau abattoir in Zamfara State Nigeria. In February 2017, a dromedary bull‐camel aged 4–6 years, slaughtered in the same facility, was found to have similar lesions. The animals were brought in from different regions of the State. Clinically, the affected animals were observed to exhibit pronounced emaciation. Prior to sample collection for laboratory investigations, affected animals were subjected to detailed meat inspection that involved visual examination, palpation and incision of suspect organs and lymph nodes conducted according to the previously described procedure (Pavlik *et al*. 2005). Caseated or calcified granulomas of various shapes and sizes were identified as bTB gross lesions. Samples for laboratory examinations were taken from organs found to have lesions; i.e. from the cow's lungs, pleura, ribs cage, diaphragm, liver, reticulo‐rumen and lymph nodes, and from the dromedary camel's lungs, intestines, liver, diaphragm and tracheobronchial lymph nodes. Collected samples were immediately transported in an icebox to the Laboratory for Microbiology at Ahmad Sani Yariman Bakura Specialist Hospital, based in Gusau Nigeria for the initial examination.

## Laboratory evaluation

A portion (about 1 g) of each sample was homogenized in 5 ml of 0.9% normal saline solution for 10 min. Thereafter, homogenates were decontaminated with 2 mL of 4% NaOH for 15 min, neutralized with 1% (0.1 N) hydrochloric acid (HCl) using phenol red as an indicator, and concentrated by centrifugation at 3000*g* for 15 min. An aliquot of the pellets from each whole‐processed sample was used to prepare a smear for acid‐fast staining and microscopy. Loops of each suspension were gently smeared on a clean, grease‐free microscopic slide, air‐dried and heat‐fixed. Ziehl–Neelsen (ZN) stain for the presence of acid‐fast bacilli (AFB) was conducted at the Laboratory for Microbiology of Ahmad Sani Yariman Bakura Specialist Hospital, based in Gusau Nigeria. The procedure of laboratory examination for acid‐fast tubercle bacilli was performed as described previously (Lumb *et al*. 2013). All samples collected from different anatomical sites of the disseminated lesions were stained.

The procedures for single‐tube multiplex‐PCR for differential identification of MTBC were conducted at the DNA‐LAB based in Kaduna, Nigeria. Only one AFB positive sample from each affected animal was examined. Genomic DNA was extracted according to the described phenol‐chloroform‐isoamyl alcohol precipitations protocol (Janice *et al*. 1997). Detected AFB were confirmed to be MTBC by RD polymerase chain reaction (PCR), described previously (Warren *et al*. 2006). Briefly, PCR amplification was performed at a final reaction volume of 20 *μ*L consisting of 1 *μ*L DNA template re‐suspended in HotStat PCR Premix (1 unit Taq DNA polymerase, 10 mmol/L Tris‐HCl (pH 9.0), 30 mmol/L KCl, 250 *μ*mol/L of each dNTPs, 1 × PCR buffer and 1.5 mmol/L MgCl_2_), 25 pmol/L of 0.5 *μ*L each of the four 3‐sequence primers (RD1, RD4, RD9 and RD12 [Table 1]) and 13 *μ*L sterile nuclease free water. Negative control mixture (Master mix) without DNA template was included in the amplification run. The reaction was carried out in PTC‐100™ Programmable Thermal Controller (MJ Research, Inc. USA) at the following conditions: initial denaturation at 95°C for 15 min followed by 45 cycles of denaturation at 94°C for 1 min, annealing at 62°C for 1 min and extension at 72°C for 1 min, with a final extension step at 72°C for 10 min. Amplification products (6 *μ*L) were separated in 3% agarose gels (Green Bio Research^®^) stained with 5 *μ*L ethidium bromide (Invitrogen^®^) at 120 V for 4 h using Horizontal Gel Electrophoresis Apparatus (GIBCOBRL Model H5‐Gaithersburg, USA). Gels were visualized, photographed and analysed by comparing band sizes between M: DNA‐Marker (100 bp Plus DNA ladder) (Bioneer, Inc. USA) and Lane A and B (samples amplified products). The band sizes observed on the gels were compared with the patterns interpreted previously by Warren *et al*. (2006) (Table 1).

**Table 1:** Target region of difference (RD), primer sequences and corresponding amplicons for the identification of species from the *M. tuberculosis* complex (MTBC)

Target RD	Primers sequence	*M*. *bovis*	*M. caprae*	References
1	AAGCGGTTGCCGCCGACCGACC	RD1 present (146 bp)	RD1 present (146 bp)	Warren *et al*. (2006)
1	CTGGCTATATTCCTGGGCCCGG			
1	GAGGCGATCTGGCGGTTTGGGG			
4	ATGTGCGAGCTGAGCGATG	RD4 absent (268 bp)	RD4 present (172 bp)	
4	TGTACTATGCTGACCCATGCG			
4	AAAGGAGCACCATCGTCCAC			
9	CAAGTTGCCGTTTCGAGCC	RD9 absent (108 bp)	RD9 absent (108 bp)	
9	CAATGTTTGTTGCGCTGC			
9	GCTACCCTCGACCAAGTGTT			
12	GGGAGCCCAGCATTTACCTC	RD12 absent (306 bp)	RD12 absent (306 bp)	
12	GTGTTGCGGGAATTACTCGC			
12	AGCAGGAGCGGTTGGATATTC			

bp, base pairs.

## Diagnosis

**Figure 1:**
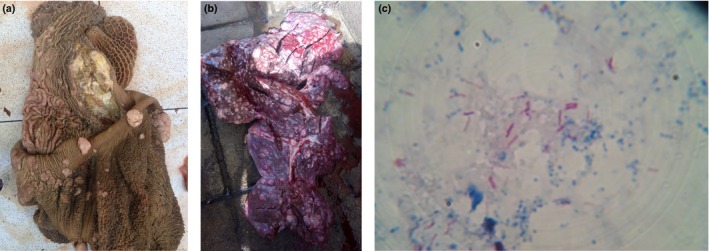
Representative of disseminated tuberculosis compatible lesions and acid‐fast bacilli (AFB) observed in a cow and a dromedary camel in Nigeria. (a) Multifocal tuberculous lesions (yellowish‐white) on reticulo‐rumen of a cow. (b) Camel's liver embedded with multiple tubercles. (c) Photomicrograph of field (×100 oil immersion); acid‐fast bacilli (AFB) appeared pinkish in a bluish background, detected in tuberculous liver from camel.

**Figure 2:**
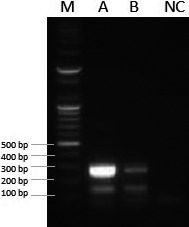
Electrophoretic fractionation of amplicons in 3% agarose. Lane M = 100 bp DNA ladder. Lane A = *Mycobacterium bovis* detected from cow with grossly disseminated TB lesions. Lane B = *Mycobacterium bovis* detected from dromedary camel with disseminated tuberculous lesions. Lane NC = negative control.

At post‐mortem examination, encapsulated foci to extensive tuberculous lesions consistent with MTBC infection were found in the cow's thoracic and abdominal cavities, and also in the pre‐mammary lymph nodes (Fig. 1a). Extensive caseous granulomas of different shapes and sizes were found in the lungs, lymph nodes, diaphragm, intestines and liver of the dromedary bull‐camel (Fig. 1b). Acid‐fast bacilli were detected in all disseminated lesions from cow and dromedary camel (Fig. 1c). However, *M*. *bovis* was identified in the representative samples from the affected cow and camel using RD deletion analysis (Fig. 2).

## Discussion

*Mycobacterium bovis* has previously been isolated from bTB‐like lesions in dromedary camels (Mamo *et al*. 2011; Zerom *et al*. 2013; Narnaware *et al*. 2015), and in cattle (Ghielmetti *et al*. 2017). Disseminated tuberculosis often results from extensive haematogenous or lymphatic spread of tubercle bacilli, due to the breakdown of a cavitating lesion (at primary foci) into the adjacent vessel. More so, rapid dissemination may occur if the pathogens gain access to serous cavities and via natural passages such as respiratory tract to gastrointestinal tract or kidney to ureter to bladder. Pleural tuberculosis referred to as ‘pearl disease’ manifests grossly inform of hard‐white tubercles that resemble cauliflower, occurring in protracted early generalization. It occurs in primary infection, derived from either the retrograde lymphatogenous spread by means of related lymphatic vessels starting from tuberculotic lymph glands (e.g. primary complex) or lung infection that follows fulminating course extending to the serosal surface. However, the fulminant form or typical acute miliary generalization is very rare in cattle. BTB lesions found in this study were similar to those previously reported in slaughtered cattle (Ahmad *et al*. 2017a,b), except for a very rare lesion discovered on reticulo‐rumen of a cow. Thus, this is the first report of bovine TB incidence that involved reticulo‐rumen in cattle in Nigeria. Lesions found in the upper part of the alimentary tract, including the ruminant four‐chambered stomach, can be seen in generalized TB, via the alimentary route infection or advanced pulmonary tuberculosis. Tuberculosis in dromedary camels is not common, but when it occurs, the generalized form is considered rare.

The deletion typing discriminates between members of the MTBC; only RD1 was intact in *M. bovis* whereas RD4, RD9 and RD12 are deleted, RD1 and RD4 are intact while RD9 and RD12 are deleted in *M. caprae* (Table 1). These findings proved evident that gross TB lesions monitored at Nigerian abattoirs may be aetiologically caused by tubercle bacilli. Cattle, certain domestic and wildlife species can act as reservoirs of *M. bovis*, serving as the primary source of infection to the susceptible hosts including camelids. While the camel is a spillover host of *M. bovis* and only becomes affected when the challenge is relatively high, acquiring the TB microbe may occur if kept in close proximity to reservoir hosts (Wernery & Kinne 2012; Ahmad *et al*. 2018). Intra‐species transmission of infection in camelids without the continuous presence of a reservoir host is uncommon.

In the present study, the TB microbe detected in dromedary camel is probably due to the consequence of the predominant production system in Nigeria. This may facilitate exposure to infection by close and repeated contact between camels and potentially infected cattle in extensively mixed transhumance. Camel TB is mainly the result of spill‐over exposure. This suggests not only the presence of high levels of infection in the environment, and a high degree of infection exchange, but also a predisposing factor at the individual animal‐level as susceptibility can be another factor. Tuberculosis in animals has important economic impacts through reduced milk and meat production as well as condemnation of affected parts or carcasses, thus affecting the well‐being of communities that rely on livestock for their livelihoods (World Health Organization, 2017).

## Conclusions

These cases illustrate *M. bovis* infection in severely emaciated slaughtered cow and dromedary camel. Although link of transmission has not been established, it is possible that infection in dromedary camel was acquired from cattle as spillover consequence of mixed extensive production commonly practised in Nigeria. From the findings in this report, the following measures should be adopted at a wider scale throughout Nigeria: prevent as much as possible a mixed production in which cattle come into repeated contact with other livestock species, improve the standards of meat inspection, while tissue samples from animals found with tuberculous lesions should be examined for the presence of tubercle mycobacteria by laboratory methods.

## Source of funding

None.

## Conflicts of Interest

None.

## Ethics statement

The authors confirm that the ethical policies of the journal, as noted on the journal's author guidelines page, have been adhered to. No ethical approval was required as this study does not involve clinical trial or experimental procedure.

## Contributions

Ibrahim Ahmad, Caleb Ayuba Kudi, Abdullahi Alhaji Magaji, Yusuf Yakubu, Zaharadden Muhammed Daninna, Samuel Shuaibu and Mansur Dandallah Salisu led the design of the study, prepared the figures and drafted the manuscript. Caleb Ayuba Kudi, Abdullahi Alhaji Magaji and Yusuf Yakubu revised the manuscript. Ibrahim Ahmad, Zaharadden Muhammad Daninna, Samuel Shuaibu and Mansur Dandallah Salisu participated in the laboratory investigations. All authors read and approved the final manuscript.
