# Supplementary material for: Disseminated tuberculosis in a cow and a dromedary bull‐camel in Zamfara State in Nigeria
Source: Vet Med Sci. 2018 Oct 25;5(1):93–8. doi: 10.1002/vms3.132 (PMC6376141; doi:10.1002/vms3.132)
Supplement: Supplementary file 1 [file VMS3-5-93-s001.pdf]

Supplementary figures

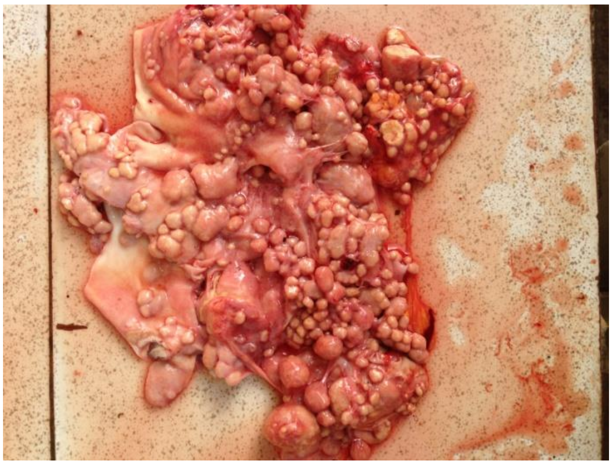

Figure 1: Diaphragm of a cow with multiple tubercles (yellowish).

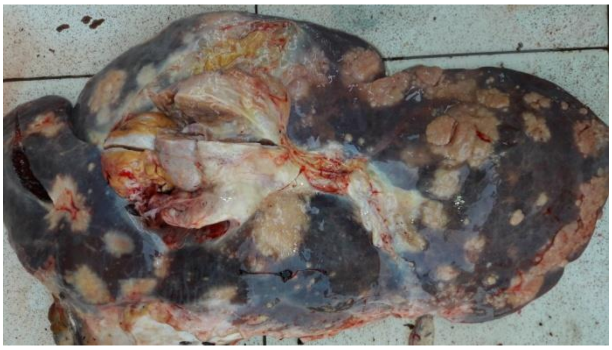

Figure 2: Enlarged tuberculous liver of a cow embedded with multiple tubercles (yellowish).

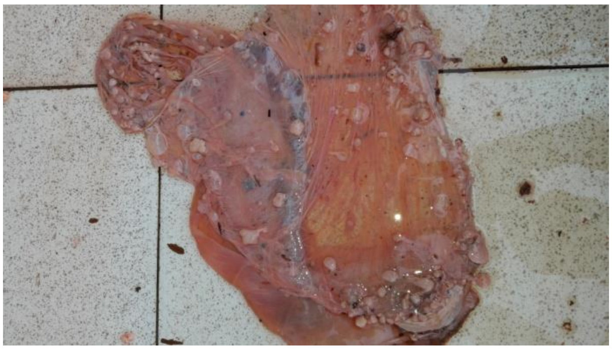

Figure 3: Pleura of a cow with tubercles (yellowish-white).

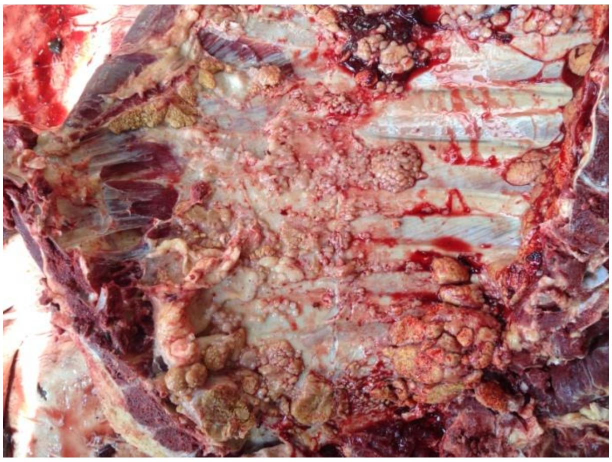

Figure 4: Rib cage of a cow covered with tubercles (yellowish colored).

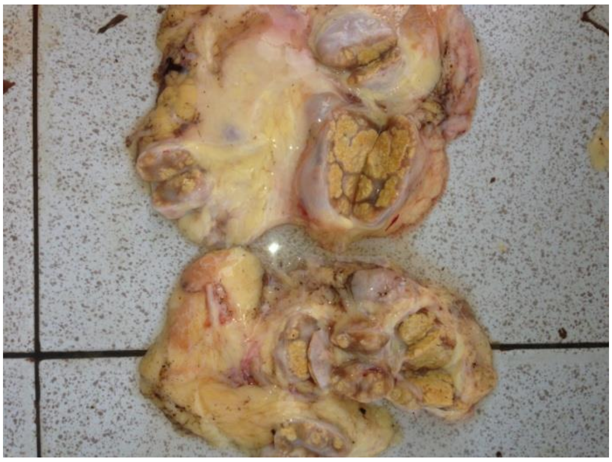

Figure 5: Incised pre-mammary lymph nodes of a cow with caseous necrosis typical of granulomatous reaction.

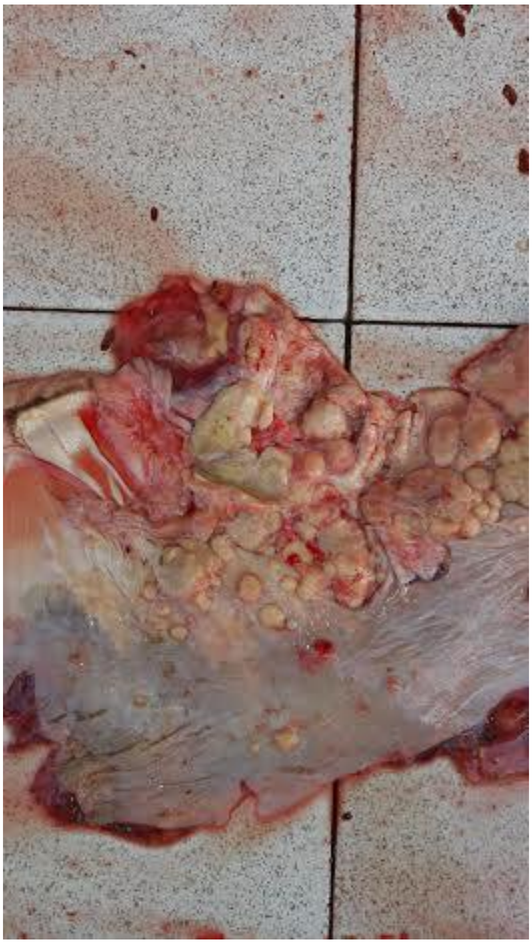

Fig. 6: Camel’s diaphragm covered with multifocal tubercles (yellowish).

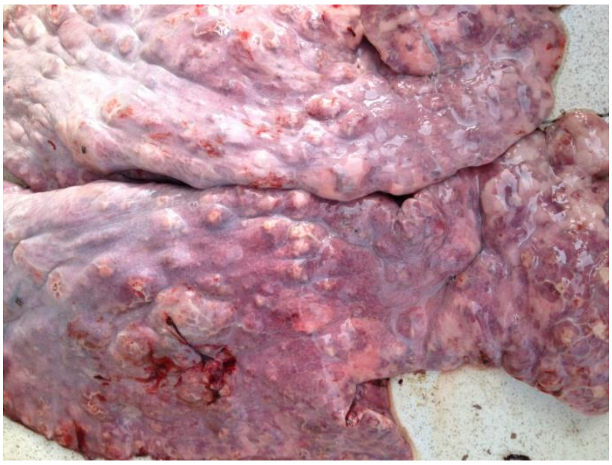

Figure 7: Camel’s lungs with multiple tubercles (grayish-white).
